# HIV Incidence among Men Who Have Sex with Men in China: A Meta-Analysis of Published Studies

**DOI:** 10.1371/journal.pone.0023431

**Published:** 2011-08-26

**Authors:** Hong-Min Li, Rui-Rui Peng, Jing Li, Yue-Ping Yin, Baoxi Wang, Myron S. Cohen, Xiang-Sheng Chen

**Affiliations:** 1 Chinese Academy of Medical Sciences Institute of Dermatology, Nanjing, China; 2 China Center for Disease Control and Prevention, National Center for STD Control, Nanjing, China; 3 Center of Infectious Diseases, University of North Carolina School of Medicine, Chapel Hill, North Carolina, United States of America; Vanderbilt University, United States of America

## Abstract

**Background:**

Men who have sex with men (MSM) have now become one of the priority populations for prevention and control of HIV pandemic in China. Information of HIV incidence among MSM is important to describe the spreading of the infection and predict its trends in this population. We reviewed the published literature on the incidence of HIV infection among MSM in China.

**Methods:**

We identified relevant studies by use of a comprehensive strategy including searches of Medline and two Chinese electronic publication databases from January 2005 to September 2010. Point estimate of random effects incidence with corresponding 95% confidence intervals (CI) of HIV infection was carried out using the Comprehensive Meta-Analysis software. Subgroup analyses were examined separately, stratified by study design and geographic location.

**Results:**

Twelve studies were identified, including three cohort studies and nine cross-sectional studies. The subgroup analyses revealed that the sub-overall incidence estimates were 3.5% (95% CI, 1.7%–5.3%) and 6.7% (95% CI, 4.8%–8.6%) for cohort and cross-sectional studies, respectively (difference between the sub-overalls, Q = 5.54, p = 0.02); and 8.3% (95% CI, 6.9%–9.7%) and 4.6% (95% CI, 2.4%–6.9%) for studies in Chongqing and other areas, respectively (difference between the sub-overalls, Q = 7.58, p<0.01). Syphilis infection (RR = 3.33, p<0.001), multiple sex partnerships (RR = 2.81, p<0.001), and unprotected receptive anal intercourse in the past six months (RR = 3.88, p = 0.007) represented significant risk for HIV seroconversion.

**Conclusions:**

Findings from this meta-analysis indicate that HIV incidence is substantial in MSM in China. High incidence of HIV infection and unique patterns of sexual risk behaviors in this population serve as a call for action that should be answered with the innovative social and public health intervention strategies, and development of biological prevention strategies.

## Introduction

The HIV epidemic in China continues to grow, expanding well beyond injection drug users (IDUs) and former plasma donors (FPDs), the first groups to suffer the spread of HIV [1]. Men who have sex with men (MSM) have now become one of the priority populations for prevention and control of HIV pandemic in China. While MSM account for only 2–4% of the Chinese adult male population [2], they comprised an estimated 32.5% of about 48,000 new HIV cases in 2009 [3]. Alarmingly, MSM represented 0.4% of patients with HIV in 2005 and 12.2% of the patients in 2007 [1], [2]. The aim of this study is to provide a comprehensive review and analysis of the scientific and empirical literature (both in English and Chinese) on the measurement of HIV incidence in Chinese MSM. In addition, we sought to identify risk factors associated with the incidence, to improve the existing programmatic and policy efforts targeting this population.

## Results

### Study inclusion

We identified 83 relevant articles in English and 24 in Chinese for evaluation. Out of these, 5 in English [4]–[8] and 6 in Chinese [9]–[14] met eligibility criteria. One article by Li et al included two independent studies reporting the incidence estimate of years 2005 and 2006, respectively [4]. One article by Han et al included three different studies from 2006, 2007 and 2008 [9], but their data from 2006 was published earlier elsewhere [10], so only data from 2007 and 2008 in Han's article were included in this analysis. The articles by Li DL et al [8] and Ruan et al [7] used the same cohort of subjects, and Li's data was used for analysis of predictors associated with HIV seroconversion, and Ruan's data was used for analysis of incidence estimates. A total of 12 studies from 11 articles consisting of more than 8000 men were included in this analysis, of which three were cohort studies [5]–[8] and nine were cross-sectional studies [4], [9]–[14]. Three studies were from north China (Beijing) [4], [7], [8], one from northwest China (Liaoning) [5], one from northwest China (Xinjiang) [13], three from east China (Jiangsu) [6], [11], [12], and four from southwest China (Chongqing) [9], [10], [14]. Characteristics of the 12 studies are present in Table 1. There was no statistically significant publication bias (Begg rank correlation test, p = 0.19), but a substantial heterogeneity among the included studies was noted (Q test, p<0.001; I^2^ = 73.58).

**Table 1:** Summarized information of the studies included in meta-analysis.

	Study year(s)	Study location	Setting^a^	Recruitment method(s)^b^	Sample size	Testing method used for estimating HIV incidence^c^	Background HIV prevalence (%)	Reported HIV incidence per 100 persons or 100 person-years
Feng LG, et al	2006	Chongqing	Internet, MSM bars, parks and bathhouses	SBR	1000	BED-CEIA	10.4	8.0
Han M, et al (1)^d^	2007	Chongqing	Internet, MSM bars, parks and bathhouses	SBR	1044	BED-CEIA	12.5	9.1
Han M, et al (2)^d^	2008	Chongqing			945	BED-CEIA	15.8	9.4
Hu HY, et al (1)^e^	2008	Jiangsu	NR^f^	NR^f^	948	RT-PCR	NR^f^	5.6
Hu HY, et al (2)^e^	2008	Jiangsu			NR^f^	BED-CEIA	NR^f^	7.5
Li SW, et al (1)^d^	2005	Beijing	Internet, MSM bars, parks and bathhouses	IBR	526	BED-CEIA	3.2	2.9
Li SW, et al (2)^d^	2006	Beijing			541	BED-CEIA	4.8	3.6
Zhang Y, et al	2007	Xinjiang	Internet	INR	143	BED-CEIA	6.5	6.7
Wang MJ, et al	2006	Chongqing	Internet, MSM bars, bathhouses, saunas, massage parlors	SBR, IBR	1000	BED-CEIA	10.3	7.3
Ruan YH, et al	2006–2008	Beijing	Internet, MSM bars, bathhouses, saunas, massage parlors	IBR, SBR	437	EIA-WB	4.8	2.6
Xu JJ, et al	2006–2007	Liaoning	NR^f^	SBR	122	EIA-WB	5.7	5.4
Yang HT, et al	2007–2008	Jiangsu	NR^f^	RDS	397	EIA-WB	4.6	5.1

a The places where the study subjects were recruited.

b SBR, snowballing recruitment; RDS, respondent-driven sampling recruitment; IBR, internet-based recruitment; and SNR, social network recruitment.

c BED-CEIA, BED capture enzyme immunoassay; RT-PCR, pooled RNA reverse transcription-PCR amplification assay; EIA-WB, enzyme immunoassay screening followed by Western blot confirmation.

d (1) and (2) represent different studies from the same article.

e (1) and (2) represent different studies from the same first author.

f NR, not reported in original paper.

### Incidence estimation

**Figure 1:**
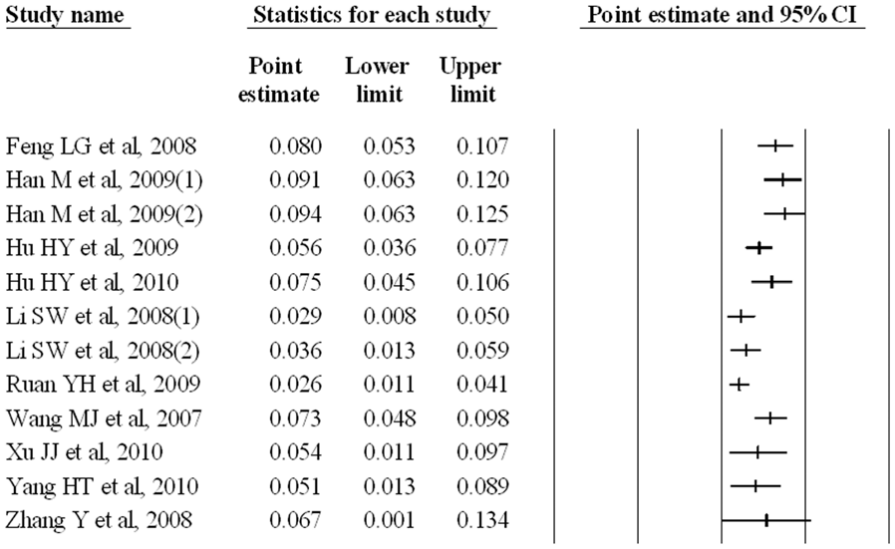
Crude results based on meta-analysis of studies assessing HIV incidence.

**Figure 2:**
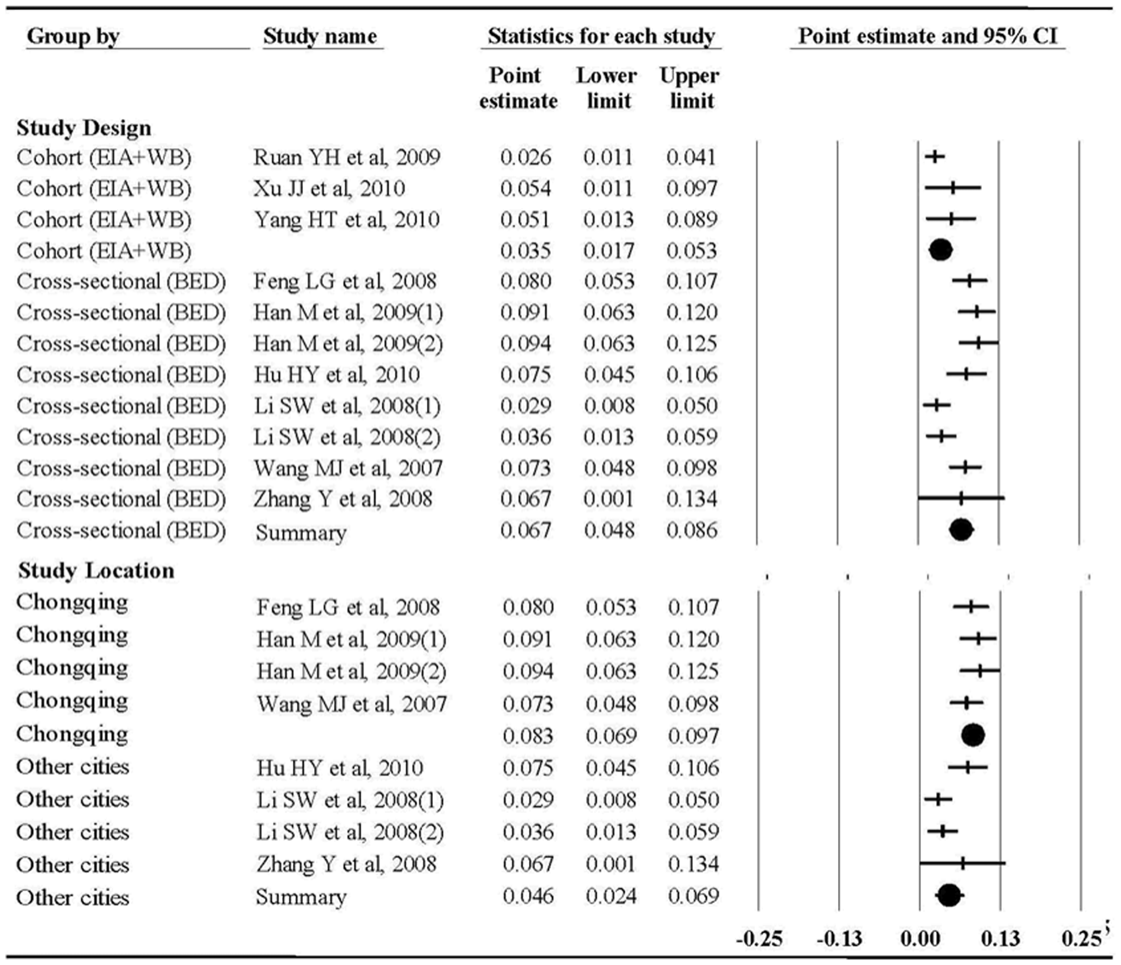
Crude results based on meta-analysis of studies assessing HIV incidence by study design, and study location.

The individual incidence rate varied from 2.6% (95% CI, 1.1%–4.1%) to 9.4% (95% CI, 6.3%–12.5%), Figure 1. The subgroup meta-analyses revealed that incidence estimates were 3.5% (95% CI, 1.7%–5.3%) and 6.7% (95% CI, 4.8%–8.6%) for cohort and cross-sectional studies, respectively (difference between the sub-overalls, Q = 5.54, p = 0.02); and 8.3% (95% CI, 6.9%–9.7%) and 4.6% (95% CI, 2.4%–6.9%) for studies in Chongqing and other areas, respectively (difference between the sub-overalls, Q = 7.58, p<0.01), Figure 2. For the subgroups, the random effects incidence estimates were nearly identical to the crude pooled incidence estimate of 3.6% (95% CI, 2.2%–5.0%) and 7.6% (95% CI, 6.4%–8.8%) for the cohort and cross-sectional studies, respectively; and 8.8% (95% CI, 7.3%–10.3%) and 4.0% (95% CI, 2.2%–5.8%) for Chongqing and other areas, respectively.

### Risk factor analysis

**Figure 3:**
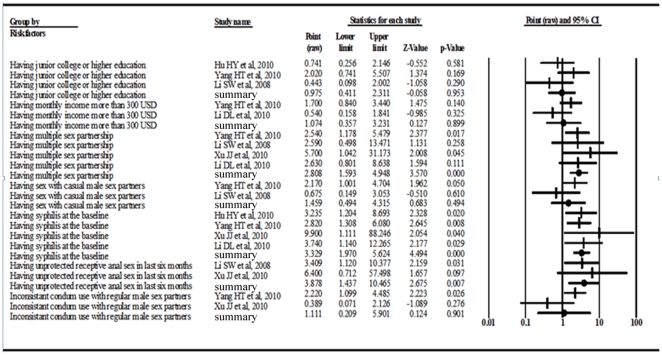
Crude results based on meta-analysis of studies assessing risk factors of HIV incidence.

Based on the five studies in which association of risk factors and incident HIV infection were reported [4]–[6], [8], [12], meta-analysis revealed that baseline syphilis infection (RR = 3.33, 95%CI, 1.97–5.62; p<0.001), multiple sex partnership (RR = 2.81, 95%CI, 1.59–4.95; p<0.001), and unprotected receptive anal intercourse in the past six months (RR = 3.88, 95%CI, 1.44–10.47; p = 0.007) were significantly associated with HIV seroconversion (Figure 3).

### Study quality

Study quality was significantly variable (Table 1). Concerns about the ability of the study sample to accurately represent the MSM population (external validity) are of concern in all studies where a convenience sample was employed. Only one of the included study [6] adopted respondent-driven sampling (RDS) recruitment, which has good representativeness, and other studies [4], [5], [7]–[10], [13], [14] included employed recruitment methods such as snowballing, social network, internet-based recruitment, and so on. Bias in detection of HIV infection (misclassification of outcome) or syphilis infection (potential risk factor) was unlikely, because assessment and confirmation of HIV or syphilis infection were conducted in the qualified laboratories using blinded methods. Information bias related to risk behaviors was obtained by self-report rather than objective measurement. The loss-to-follow-up was greater than 40% in one of 3 cohort studies [5], and participation and completeness rates were not reported or unclear in most of the cross-sectional studies, resulting in a significant attrition bias. Statistical adjustments for measured confounding factors were made in some of the included studies. Cross-sectional studies employed laboratory methods, such as BED assay and pooled RNA reverse transcription PCR (PT-PCR) amplification assay, to measure HIV incidence. However, as the dominating employed laboratory method in China, BED assay may overestimate the number of incident cases mainly because of probable misclassification of long term infection as recent HIV infection [15]–[18].

## Discussion

MSM transmission of HIV is a critical and growing public health problem in China. The HIV seroprevalence rate among MSM has been reported to be 2.5% (95% CI 0.9% to 3.3%) [19]. A national study survey of more than 18,000 MSM in 61 Chinese cities in 2008 reported an HIV prevalence of 4.9%, varying from over 10% in the south-west, to 7% in the east and 4–5% along coast in the south and north-east [20]. The empirical prevalence of HIV 4.9% in 2008 is much higher than derived from 26 studies published during 2001–2008 [19].

Since the late 1990s, increasing numbers of newly diagnosed HIV infections in MSM have been observed in many countries with large and visible MSM communities [21]. Segura et al reported an incidence of 3.9% (95% CI, 2.0%–6.7%) among MSM in Buenos Aires in a cohort study during 2003 to 2004 [22]. Hurtado et al observed an increase in incidence from 2002 to 2003 in this population in Valencia, Spain [23].

Since the nationwide prospective cohort studies are not possible to include in the national surveillance program, regular meta-analyses based on the improved data from cross-sectional and prospective studies may provide important information for developing and monitoring the intervention programs, although the results from the current meta-analysis were not able to exactly represent the HIV incidence among MSM in China considering the limitations in estimation method, sampling strategy, number of studies and sample size, representativeness and coverage of rural areas, etc. With a reasonable geographic coverage consisting of Jiangsu (east China), Beijing (north China), Liaoning (northeast China), Chongqing (southwest China), and Xinjiang (northwest China), reasonable comparability of BED and prospective cohort study in estimation of incidence, and moderate quality of the studies included, the findings from our meta-analysis could a glance of HIV incidence among the particular subgroup in China. In this meta-analysis, we included 12 studies from 11 articles publishing in English or Chinese and covering 5 regions in China, making this the first systematic and comprehensive review of HIV incidence to date. The individual incidence of HIV infection included in this meta-analysis varied widely from 2.6% to 9.4%. The observed disparity reflects differences in study methods and study locations.

The incidence of HIV infection among MSM is much higher than the reports in heterosexual populations. Studies in Yunnan, one of the provinces worst hit by HIV in China, reported the incidence estimates of 5.5%, 1.4%, 0.1%, and 0.2% amongst the discordant couples, female sex workers, pregnant women, and pre-marital couples, respectively [24]. HIV incidence among sexually transmitted disease clinic patients was reported to be 0.04% (95% CI, 0.02% to 0.10%) in Guangxi of China [25].

Chongqing is the largest municipality in southwest China, adjacent to Sichuan and Guizhou, provinces most affected by intravenous transmission of HIV by drug use. Chongqing has a higher HIV prevalence than the national average [26]. The increased incidence of HIV in MSM in Chongqing might reflect the combined risk from unprotected sex and unsafe injections. The results of our risk factor meta-analysis showed that baseline syphilis infection, multiple sex partnership, and unprotected receptive anal intercourse in the past six months were significantly associated with HIV seroconversion, which reflects the ‘unsafe sex’ among MSM. Such associations are consistent with findings by Xiao et al [27] and van der Bij et al [28], and offer direction for HIV prevention and control programmes. Behaviors of illegal drug use among MSM are not well known although a range of 0.1–44% was reported [29], since some MSM might be afraid of identifying themselves as IDUs. One of the included studies [5] reported that 2.8% of baseline MSM population had illegal drug use history in Shenyang. Yang X, et al reported 6.5% (65/1000) of recruited MSM had drug use history in Chongqing [30], which is much higher than other districts, and among 65 MSM who ever had drug use behavior, four ever had injection drug use in the last six months, and two admitted to have shared needles. In addition, southwest China is a region with higher HIV transmission through drug use than many other regions in China. MSM/IDU should be a dual-risk in this sub-population for HIV infection. Further studies are needed to investigate drug use behavior and needle sharing behavior among MSM to clarify the dual risk of HIV infection this subgroup.

Study designs may affect results. According to the result of our subgroup meta-analysis by study design, the cohort studies cited [5]–[7] had lower HIV incidence than cross-sectional studies [9]–[14]. Prospective cohort studies accurately estimate HIV incidence if seroconversion is observed, but this method is susceptible to recruitment bias, loss to follow-up, short duration of follow-up, inclusion of essential prevention intervention(s) that modify the results, and other unanticipated factors. Cross-sectional studies have employed laboratory methods, mainly BED assays in China, to measure HIV incidence. However, the accuracy of the BED results has been extensively reviewed, and it may vary by place, time and age [18], or depend on the population sampled and storage of specimens [27]. Misclassification of BED, which can lead to overestimated HIV incidence, mainly include HIV-infected individuals under antiretroviral therapy, patients with advanced immunodeficiency, and different HIV subtypes [15], [16], [31]. However, in China many of studies on BED-based incidence estimation were conducted on surveillance samples in which the complicating factors might not be common [32], [33]. Although earlier studies indicated that the performance of BED assay on HIV-1 subtype C-infected individuals was questionable, Parekh et al [34] reported that the HIV-1 BED assay worked well with subtype C. HIV infection with subtype C has been observed among heterosexual contacts in Asian country [35]. It may be needed to evaluate the performance of BED assay in estimation of incidence for HIV-1 subtype C in China. Based on the ability of BED to discriminate recent from long-term seroconversion of HIV-1 infection among MSM, further molecular analyses can be possible to investigate the distribution of subtypes and monitor the genetic variation of the HIV epidemic in China and among MSM.

Social stigma in China makes the MSM population very hard to reach. Subjects were recruited for study from internet or MSM venues [4], [7], or through snowball sampling or respondent driven sampling [6], [9], [10]. Out of 3 cohort studies one retained less than 60% [5], while another retained near 90% of the initial sample at the 12-month follow up [7]. MSM with high risk behaviors may not choose to participate in HIV monitoring programs [36]–[38]. It has been previously reported that cohort participants who returned regularly for follow-up visits were significantly less likely to report high-risk behaviors compared with those who are lost to follow-up [39]. Ruan et al reported that men who had a higher level of education were more likely to retain in the cohort [7]. It was reported from previous studies in China that more than one-third of the MSM had ever been married, and more than 70% of well-educated gays or bisexuals had got married or would marry with women [40]–[42]. Choi et al [43] showed that 28% of MSM in Beijing self-reported to ever have sex with both men and women during the last six months. Zhang et al [44] reported that 63.6% of MSM in China ever had casual male sex partners, and 50% of MSM ever had sex with women in the last year, and the high risk behaviors among this population implies that MSM might be bridge population for others for HIV transmission.

Several biomedically based interventions for prevention of sexual transmission of HIV have obtained encouraging outcomes while many others are currently under way with biological and clinical investigations. Studies in several countries have shown that daily administration of two oral antiretroviral drugs, emtricitabine and tenofovir disoproxil fumarate could applied provide a significant protection against the acquisition of HIV infection by 44% among MSM [45]. This preexposure chemoprophylaxis may provide an opportunity for HIV prevention among this population, but there are still a lot of considerations or concerns in translating this evidence into intervention strategy, particularly in developing countries. An antiretroviral-based vaginal microbicide has proved reduction of HIV acquisition by 39% in women in a recent randomised controlled clinical trial in South Africa [46], but rectal microbicides remain at early stages of clinical investigation [47]. In order to curb the increasing epidemic of HIV among MSM in China, more risk-reduction intervention efforts are in urgent need and these intervention efforts should be guided by cultural and social context and be responsive to unique demographic characteristics and risk profiles of different subgroups [29]. Health education and behavioral interventions are still primary prevention measures in order to reduce the risk-taking behaviors mainly involved in anal intercourse. More innovative and structural interventions including HIV testing and counseling, treatment of other STIs, integration of STI and counseling and testing services into prevention activities remain a focus of prevention and control efforts to MSM in China.

It is concluded that HIV incidence is high in MSM populations in China, and the high risk behaviors among this population implies that MSM might be bridge population for others for HIV transmission. It is assumed that MSM/IDUs are dual-risky for HIV infection, but further researches are needed. The 2007–2010 Guidelines for Prevention and Control of HIV/AIDS among Men Who Have Sex with Men includes a range of evidence-based HIV preventive measures, but few interventions have proven benefit in study with appropriate design and/or implementation, in China or elsewhere [48]. The considerable incidence of HIV infection and unique patterns of high risk behaviors in MSM in China serve as a call for action that must inspire new and innovative social and public health HIV prevention strategies. More studies may be necessary in China in future research, at least including (1) validation of cross-sectional BED assay to estimate HIV-1 incidence among patients on antiretroviral therapy and with advanced disease, and other subgroups as well; (2) validation of cross-sectional BED assay for subtype C of HIV-1; (3) development of modeling methods more appropriate for estimating HIV-1 incidence in Chinese settings; and (4) application of BED assays on molecular epidemiological studies of HIV-1 infection.

## Materials and Methods

### Study identification

**Figure 4:**
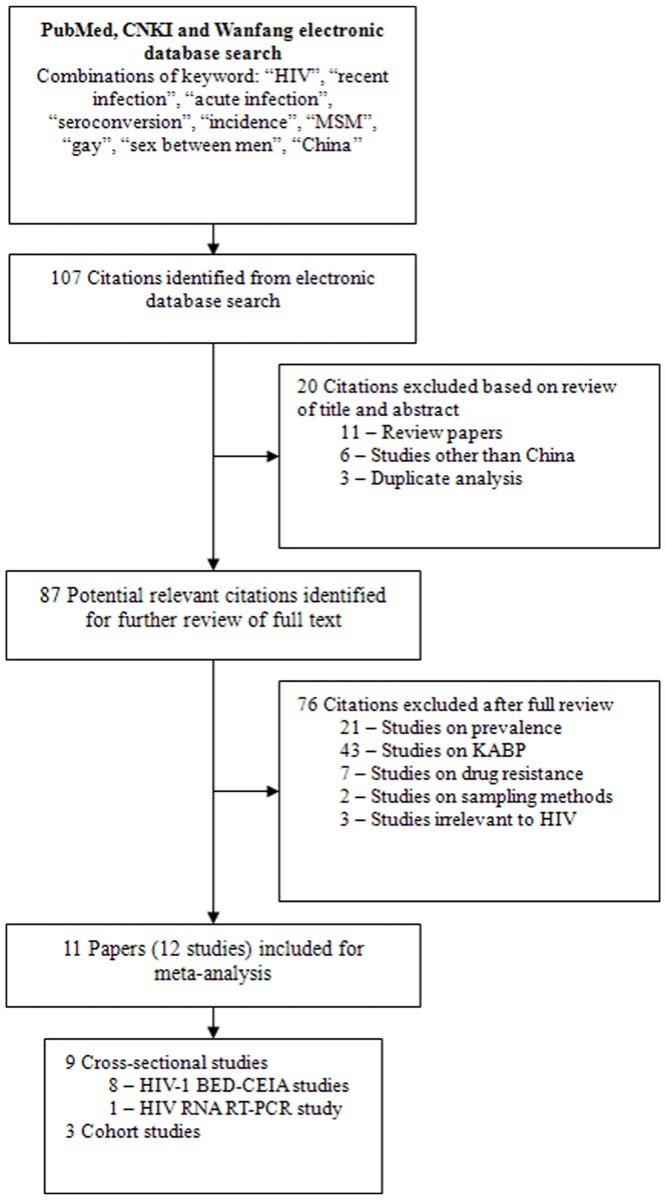
Identification, review, and selection of studies included in the meta-analysis.

Identification of relevant studies was carried out by 3 of the authors (HML, JL and RRP), who comprehensively searched PubMed-MEDLINE, China National Knowledge Information (CNKI) and Chinese Wanfang databases for articles published between January 2005 and September 2010. The search strategies of combining the following key words in English and their corresponding terms in Chinese were used: HIV, recent infection, acute infection, seroconversion, incidence, MSM, gay, sex between men, and China. The review of papers was conducted in two stages (Figure 4). Title and abstract review of all searched articles was completed by 2 of the authors (HML and JL). Full reports of 87 potentially relevant articles were independently reviewed by 2 of the authors (HML and RRP) to include the articles for the meta-analysis. To ensure efficiency of the search, reference lists of the articles from the archives were also examined.

Studies that met each of the following criteria were considered eligible: (1) those estimating incidence of HIV-1 infection; (2) those with clear descriptions of study design, testing method to determine HIV infection, study location within China, and sample size; (3) those conducted among Chinese MSM; and (4) those published in English or Chinese. The criteria for exclusion are as follows: (1) those other than on HIV; (2) those not determining HIV incidence, including those related to knowledge, attitude, behavior and perception (KABP), drug resistance, or prevalence of HIV; (3) those from non-original studies; or (4) those conducted in other populations or other countries. If a study was reported in duplicate, the article published in English or published earlier or earliest was included in the analysis. Disagreements on eligibility between the reviewers were resolved through discussion to meet consensus.

### Data extraction

A standardized data extraction form was used for recording information including study year, location, sample size, study design, sample recruitment method, testing algorithm used to estimate the incidence, background HIV prevalence, and reported HIV incidence of each included study (Table 2). Data extraction was completed by 2 of the authors (HML and RRP).

**Table 2:** Quality assessment of the studies included in meta-analysis.

Study	External validity		Internal validity											
	Represen- tativeness^a^	Sample size^b^	Partici- pation rate^c^	HIV test^d^	Com- plete- ness^e^	Reten- tion rate^f^	Follow- up dura- tion^g^	Loca- tion	Self-report					STI test^h^
									Age	Marital status	Drug use	Condom use	Other exposure	
Cross-sectional studies														
Feng LG, et al	…	✓	NR	✓	NR	NA	NA	✓	…	…	…	…	…	…
Han M, et al (1)^i^	…	✓	NR	✓	NR	NA	NA	✓	…	…	…	…	✓	…
Han M, et al (2)^i^	…	✓	NR	✓	NR	NA	NA	✓	✓	…	…	…	✓	…
Hu HY, et al (1)^j^	…	✓	NR	✓	NR	NA	NA	✓	…	…	…	…	…	…
Hu HY, et al (2)^j^	…	✓	NR	✓	NR	NA	NA	✓	✓	✓	…	✓	✓	✓
Li SW, et al (1)^i^	…	✓	NR	✓	✓	NA	NA	✓	✓	…	…	✓	✓	…
Li SW, et al (2)^i^	…	✓	NR	✓	NR	NA	NA	✓	✓	…	…	✓	✓	…
Zhang Y, et al	…	…	✓	✓	NR	NA	NA	✓	…		…	…	…	✓
Wang MJ, et al	…	✓	NR	✓	NR	NA	NA	✓	✓	…	…	…	…	…
Cohort studies														
Ruan YH, et al	…	✓	✓	✓	NA	✓	✓	✓	✓	✓	✓	✓	✓	✓
Xu JJ, et al	…	…	✓	✓	NA	…	✓	✓	✓	✓	✓	✓	✓	✓
Yang HT, et al	✓	…	✓	✓	NA	✓	✓	✓	✓	✓	…	✓	✓	✓

Symbol ✓ indicates the measure was adequately addressed in the study; NR = not report; NA = not applicable.

a Studies received a ✓ if the sample included all eligible HIV-negative men over a defined time period and defined area, or a random or systematic sample of those men (e.g., RDS);

b Studies received a ✓ if the sample size was 456 or more which is the sample size required to estimate the incidence given α = 0.05, δ = 0.02, and expected rate = 5%.

c Studies received a ✓ if the percentage participation was 80% or more;

d Studies received a ✓ if the outcome (HIV infection) was based on blinded laboratory testing;

e Studies received a ✓ if the percentage participants in the final analysis was 80% or more for cross-sectional studies;

f Studies received a ✓ if the percentage participants under successful retention when the outcome was evaluated was 70% or more for cohort studies;

g Studies received a ✓ if the follow-up duration was 6-month or more for cohort studies;

h Studies received a ✓ if the identification of STI was based on laboratory testing rather than self-report.

i (1) and (2) represent different studies from the same article.

j (1) and (2) represent two different articles from the same first author.

The report included were categorized according to study design (cross-sectional study; cohort study) with corresponding testing method (BED capture enzyme immunoassay, BED-CEIA; pooled reverse transcription-PCR amplification assay, RT-PCR; enzyme immunoassay screening followed by Western blot confirmation, EIA-WB), and study location (Chongqing and other areas).

### Quality assessment

A set of items (Table 1) for assessing the methodological quality of the articles was used for cross-sectional and cohort studies, respectively. The items included two separate sections to appraise external and internal validity, including biases relevant to data collection and classification in the studies. Two of the authors independently evaluated study quality, and disagreement was resolved by discussion with a third reviewer.

### Statistical analysis

The reported incidence estimates were calculated as the ratios of the numbers of serconverters divided by the person-years of follow-up for cohort studies, and the maximum likelihood estimates using McDougal formula for sensitivity/specificity adjustments and Hargrove formula for specificity adjustments for cross-sectional studies [39], [49]. Weighted averages of reported incidences based on study size were calculated as crude pooled incidence statistics. We did not conduct pooled analyses as we could not get original data from the eligible papers. Point estimate of incidence with corresponding 95% confidence intervals (CI) of HIV infection were carried out using the Comprehensive Meta-Analysis software (CMA, version 2.0, Biostat Inc., Englewod, NJ, USA). Subgroup analyses based on study design and geographic location were examined separately.

Heterogeneity between studies was tested with the Q test (p<0.10 indicating a statistically significant heterogeneity) and the I^2^ statistic (larger values showing the increasing heterogeneity, with 25% as low, 50% as moderate and 75% as high heterogeneity between studies) [50]. If the data are heterogeneous, random effects models were used for meta-analysis. The Begg rank correlation method was used to assess the potential for publication bias (p<0.05 indicating a statistically significant publication bias).

## References

[1] State Council AIDS Working Committee Office, UN Theme Group on AIDS in China (2007). A Joint Assessment of HIV/AIDS Prevention, Treatment and Care in China.

[2] Zhang B, Li X, Shi T (2002). A primary estimation of the number of population and HIV prevalence in homosexual and bisexual men in China.. Chin J AIDS/STD Prev Control.

[3] Ministry of Health. China 2010 UNGASS Country Progress Report.. http://data.unaids.org/pub/Report/2010/china_2010_country_progress_report_en.pdf.

[4] Li SW, Zhang XY, Li XX, Wang MJ, Li DL (2008). Detection of recent HIV-1 infections among men who have sex with men in Beijing during 2005–2006.. Chin Med J (Engl).

[5] Xu JJ, Zhang M, Brown K, Reilly K, Wang H (2010). Syphilis and HIV seroconversion among a 12-month prospective cohort of men who have sex with men in Shenyang, China.. Sex Transm Dis.

[6] Yang HT, Hao C, Huan X, Yan H, Guan W (2010). HIV incidence and associated factors in a cohort of men who have sex with men in Nanjing, China.. Sex Transm Dis.

[7] Ruan YH, Jia Y, Zhang X, Liang H, Li Q (2009). Incidence of HIV-1, syphilis, hepatitis B, and hepatitis C virus infections and predictors associated with retention in a 12-month follow-up study among men who have sex with men in Beijing, China.. J Acquir Immune Defic Syndr.

[8] Li DL, Jia YJ, Ruan YH, Liu Y, Li Q (2010). Correlates of incident infections for HIV, syphilis, and hepatitis B virus in a cohort of men who have sex with men in Beijing.. AIDS Patient Care STDS.

[9] Han M, Feng LG, Jiang Y, Shen S, Ling H (2009). Surveillance on HIV-1 incidence among men who have sex with men in Chongqing, China, 2006–2008.. Chin J Epidemiol.

[10] Feng LG, Wang MJ, Han M, Ding XB, Jiang Y (2008). Drug resistance among recent HIV-1 infected men who have sex with men in Chongqing municipality of China.. Chin J Epidemiol.

[11] Hu HY, Guo HX, Fu GF, Xu XQ, Huan XP (2009). Application of pooled HIV-1 RNA RT-PCR in detecting HIV window period in MSM.. Acta Univ Med Nanjing.

[12] Hu HY, Shen S, Huan XP, Yan HJ, Xiao Y (2010). Estimation of HIV-1 incidence of MSM in Jiangsu Province with BED-CEIA assay.. Acta Univ Med Nanjing.

[13] Zhang Y, Liu JW, Ni MJ, Dong YH (2008). Laboratory study on detective results of male homosexuality population in Urumqi, Xinjiang.. Endemic Dis Bull.

[14] Wang MJ (2007). Research on BED-CEIA in detecting recent infections and on drug resistance in recent HIV-l infections in Dehong prefecture and Chongqing Municipal.. Thesis (in Chinese).

[15] Laeyendecker O, Rothman RE, Henson C, Horne BJ, Ketlogetswe KS (2008). The effect of viral suppression on cross-sectional incidence testing in the johns hopkins hospital emergency department.. J Acquir Immune Defic Syndr.

[16] Joint United Nations Programme on HIV/AIDS (UNAIDS) (2005). UNAIDS Reference Group on Estimates, Modelling and Projections' statement on the use of the BED-assay for the estimation of HIV-1 incidence for surveillance or epidemic monitoring.. http://data.unaids.org/pub/EPISlides/2006/statement_bed_policy_13dec05_en.pdf.

[17] Hargrove JW, Humphrey JH, Mutasa K, Parekh BS, McDougal JS (2008). Improved HIV-1 incidence estimates using the BED capture enzyme immunoassay.. AIDS.

[18] Hallett TB, Ghys P, Bärnighausen T, Yan P, Garnett GP (2009). Errors in ‘BED’-derived estimates of HIV incidence will vary by place, time and age.. PLoS One.

[19] Gao L, Zhang L, Jin Q (2009). Meta-analysis: prevalence of HIV infection and syphilis among MSM in China.. Sex Transm Infect.

[20] Anonymous Men who have Sex with Men (MSM) – Update for ICAAP, Bali, 2009: the 2008 Chinese Nation-wide survey of 18,101 MSM.. http://www.msmasia.org/tl_files/news/ICAAP_News/China_MSM_Country_Snapshot%20_Aug_2009.pdf.

[21] Marcus U, Voss L, Kollan C, Hamouda O (2006). Surveillance report, HIV incidence increasing in MSM in Germany: factors influencing infection dynamics.. Euro Surveill.

[22] Segura M, Sosa Estani S, Marone R, Bautista CT, Pando MA (2007). Buenos Aires cohort of men who have sex with men: prevalence, incidence, risk factors, and molecular genotyping of HIV type 1.. AIDS Res Hum Retroviruses.

[23] Hurtado I, Alastrue I, Ferreros I, del Amo J, Santos C (2007). Trends in HIV testing, serial HIV prevalence and HIV incidence among people attending a Center for AIDS Prevention from1988 to 2003.. Sex Transm Infect.

[24] Duan S, Shen S, Bulterys Marc, Jia Y, Yang Y (2010). Estimation of HIV-1 incidence among five focal populations in Dehong, Yunnan: a hard hit area along a major drug trafficking route.. BMC Public Health.

[25] Yin YP, Chen XS, Wang HC, Shi MQ, Wei WH (2008). Detection of acute HIV infections among sexually transmitted disease clinic patients: a practice in Guangxi Zhuang Autonomous Region, China.. Sex Transm Infect.

[26] Feng L, Ding X, Lu R, Liu J, Sy A (2009). High HIV prevalence detected in 2006 and 2007 among men who have sex with men in China's largest municipality: an alarming epidemic in Chongqing, China.. J Acquir Immune Defic Syndr.

[27] Xiao Y, Ding X, Li C, Liu J, Sun J (2009). Prevalence and correlates of HIV and syphilis infections among men who have sex with men in Chongqing Municipality, China.. Sex Transm Dis.

[28] van der Bij AK, Stolte IG, Coutinho RA, Dukers NH (2005). Increase of sexually transmitted infections, but not HIV, among young homosexual men in Amsterdam: are STIs still reliable markers for HIV transmission?. Sex Transm Infect.

[29] Guo Y, Li X, Stanton B (2011). HIV-related behavioral studies of men who have sex with men in China: a systematic review and recommendations for future research.. AIDS Behav.

[30] Yang X, Yi D, Ding XB (2008). Status of high risk behavior and infection of HIV/AIDS among 1000 men who have sex with men in a city.. Acta Academiae Medicinae militaris tertiae.

[31] Bärnighausen T, McWalter TA, Rosner Z, Newell ML, Welte A (2010). HIV incidence estimation using the BED capture enzyme immunoassay: systematic review and sensitivity analysis.. Epidemiology.

[32] Xiao Y, Jiang Y, Feng J, Xu W, Wang M (2007). Seroincidence of recent human immunodeficiency virus type 1 infections in China.. Clin Vaccine Immunol.

[33] Xu J, Wang H, Jiang Y, Ding G, Jia M (2010). Application of the BED capture enzyme immunoassay for HIV incidence estimation among female sex workers in Kaiyuan City, China, 2006–2007.. Int J Infect Dis.

[34] Parekh BS, Hu DJ, Vanichseni S, Satten GA, Candal D (2001). Evaluation of a sensitive/less-sensitive testing algorithm using the 3A11-LS assay for detecting recent HIV seroconversion among individuals with HIV-1 subtype B or E infection in Thailand.. AIDS Res Hum Retroviruses.

[35] Praparattanapan J, Tragoolpua Y, Pathom-Aree W, Kotarathitithum W, Chaiwarith R (2011). Current molecular epidemiology and recombination of HIV type 1 subtypes in Northern Thailand.. AIDS Res Hum Retroviruses 2011 May 6.

[36] Feng Y, Wu Z, Detels R (2010). Evolution of men who have sex with men community and experienced stigma among men who have sex with men in Chengdu, China.. J Acquir Immune Defic Syndr.

[37] Neilands TB, Steward WT, Choi KH (2008). Assessment of stigma towards homosexuality in China: a study of men who have sex with men.. Arch Sex Behav.

[38] Qian HZ, Vermund SH, Wang N (2005). Risk of HIV/AIDS in China: subpopulations of special importance.. Sex Transm Infect.

[39] Anonymous BED-CEIA Incidence and Adjustment Formula.. http://www.calypte.com/pdf/Adjustment-Formula.pdf.

[40] Lau JT, Wang M, Wong HN, Tsui HY, Jia M (2008). Prevalence of bisexual behaviors among men who have sex with men (MSM) in China and associations between condom use in MSM and heterosexual behaviors.. Sex Transm Dis.

[41] He Q, Wang Y, Lin P, Liu Y, Yang F (2006). Potential bridges for HIV infection to men who have sex with men in Guangzhou, China.. AIDS Behav.

[42] Zhang BC, Chu QS (2005). MSM and HIV/AIDS in China.. Cell Res.

[43] Choi KH, Gibson DR, Han L, Guo Y (2004). High levels of unprotected sex with men and women among men who have sex with men: a potential bridge of HIV transmission in Beijing, China.. AIDS Educ Prev.

[44] Zhang BC, Li XF, Shi TX, Cao NX, Hu TZ (2002). Survey on high risk behaviors and other AIDS/STI related factors among men who have sex with men (MSM) in mainland China ('2001).. Chin J Dermatol.

[45] Grant RM, Lama JR, Anderson PL, McMahan V, Liu AY (2010). Preexposure chemoprophylaxis for HIV prevention in men who have sex with men.. N Engl J Med.

[46] Abdool Karim Q, Abdool Karim SS, Frohlich JA, Grobler AC, Baxter C (2010). Effectiveness and safety of tenofovir gel, an antiretroviral microbicide, for the prevention of HIV infection in women.. Science.

[47] Beyrer C (2010). Global prevention of HIV infection for neglected populations: men who have sex with men.. Clin Infect Dis.

[48] Padian NS, McCoy SI, Balkus JE, Wasserheit JN (2010). Weighing the gold in the gold standard: challenges in HIV prevention research.. AIDS.

[49] Brookmeyer R, Quinn T, Shepherd M, Mehendale S, Rodrigues J (1995). The AIDS epidemic in India: a new method for estimating current human immunodeficiency virus (HIV) incidence rates.. Am J Epidemiol.

[50] Higgins JP, Thompson SG, Deeks JJ, Altman DG (2003). Measuring inconsistency in meta-analyses.. BMJ.

